# The Crime of Love

**Published:** 1911-05

**Authors:** G. Henri Bogart

**Affiliations:** Brookville, Ind.


### Abstracts and Selections.

### The Crime of Love

G. HENRI BOGART, M. D., BROOKVILLE, IND.

A bride at the nuptial couch has a great, aye, immeasurably a greater right to demand previous chastity and continence of her spouse than he has to exact a like standard of virtue from her. I am not advancing this thought to shock the reader, nor yet to precede an assault on conventional relations of the sexes, but simply as a pathological and prophylactic proposition.

Marriage means a sexual partnership with whatever else of mutuality that the wisdom of the partners, or their whims, may connect or devise, and the more the elements of their union the stronger the partnership.

When a partner comes into a firm with his principal asset bankrupt, or mortgaged to some secret claim, and conceals the fact from the other partner, or, worse yet, when he comes with that demand which shall hang a perpetual drain upon the original resources of the other, we would send the guilty one to the penitentiary for fraud, and yet we observe the results of exactly these things in the marriage contract every day and accept it as a matter of course.

The larger proportion of the women who are sent to the surgeon's table for abdominal and sexual surgery are sent there by the action of specific infection, innocently derived from supposedly cured husbands. Morrow places the percentage as high as 80, while a writer in *Pearson's Magazine* claims that 65 per cent of the women who submit to pelvic operation at Johns Hopkins, that most exclusive of hospitals for the elite of the South, come there as the result of negro prostitution. While I do not believe that in these cases it is possible to secure the absolute figures, we all know that the number is very great, and that there should be any is an outrage upon the race.

Now, for another point: Professor Irvine Fisher, of Yale, in his elaborate report on Vital Statistics to the National Conservation Commission, shows that for all the United States registered reporting area the death percentage from obscure heart, brain and kidney diseases has increased 131 per cent since 1880 and that 80 per cent of this increase has affected the male. Almost every practician has encountered cases of heart, brain and kidney disease which would not yield any satisfactory diagnosis nor respond to any routine treatment, and more recently the microscope has shown the gonococci swarming in these organs in such cases, when no other means would determine the cause of death; and this increase exactly parallels the development of constitutional gonorrhea.

In my own service as coroner for fourteen years I have encountered cases in which the ordinary autopsy did not disclose the cause of death, while the microscope did. A specimen case will fully demonstrate my meaning. A lady came to our home to visit. Fourteen years before, she, a young widow, had married. A few months after her second marriage her health failed, as did her husband's. For fourteen years the two have been the feeders for the fakers, she for "female trouble," he for "stomach trouble," and as he made good wages they were a couple of veritable little gold mines. Not the fakers alone had profited, for they had run the entire gamut of medical service, and all to no avail. She had reached that state of querulous, neurasthenic invalidism wherein any conversation soon became an "organ recital." She told me of her ailments, ad nauseam, and of what her various attendants had told her, and really the woman had lived in a veritable hell, her pregnancies had aborted, and now she was unsexed, for, as she put it, "It might as well be my apron pocket as anywhere else."

I learned that her husband was for the time under the care of a capable, reputable physician, and so I opened communication with him and told him what I suspected. Bacteriological investigation showed the husband infected, without any of the ordinary indications, he was a gonococcus carrier. He told a story of having been "burnt" a couple of years before marriage, and related that as soon as he discovered that he had a "dose" he had gone to the best "specialist in the city," a cheap druggist, who had dried it up in a few days, and only charged him a nominal price. He persisted in telling how slight an affair gonorrhea is, and told that he had sent many friends to the druggist.

Proper treatment, administered without his cognizance of the real ailment, has largely relieved his condition, though with his wife the damage is beyond repair. This is one of the countless thousands of domestic tragedies too often unrecognized. It is estimated that four-fifths of all young men contract gonorrhea, and though many of these only suffer from the simpler form and escape the constitutional contamination, yet the whole matter is so little understood that no man who has been exposed to infection should marry until competent bacteriological investigation has shown him free from the germs of Neisser.

Even today many physicians are content with merely drying up the discharge and relieving the painful symptoms, then turning the patient loose; too many treat the whole matter as a nasty joke, and others, while acknowledging the gravity, excuse themselves for allowing these patients to go by, saying that they will not "stand for" constitutional treatment and examinations. This latter I do not believe.

If the matter be rightly explained to the average man, he would lose his right hand rather than visit this hellish fate upon the woman whom he loves. Here in Indiana we have a forty-page pamphlet, issued by the State Board of Health, now in its sixtieth thousand, in which these facts are set forth with startling plainness for the laity.

What we need in this matter is publicity, intelligent publicity, which, while scorning the silliness of prudery, shall at the same time avoid offensive and unnecessary salacious thought. It is useless for us to say that we shall do away with prostitution, and equally useless for us to say that we shall keep all the young men from its embraces, for this "the oldest trade in the world" has existed among all peoples and in all climes.

We must meet facts as they are and not as we would have them. The ostrich which hides its head in the sand is the favorite type of absurd cowardice, and how much better are the masses of our people when they are considering this momentous question, and allowing false ideals to chain us down, with the damning falsehood, that "ignorance is innocence."

To the doctor I will now drop a hint as to treatment. The use of arsenic sulphide and calcium sulphide, exhibited jointly, and pushed to the point of saturation, seems as specific for the destruction of gonococci, as is mercury for syphilis, and the treatment is rational, since the two active elements of this treatment are of the most active of the germicides, capable of systemic toleration to the point of germicidal activity.

Our double standard of morality, which expects a young man to debauch himself sexually and winks a doubting smile, when he claims continence for himself, while it contemplates absolute purity in his sister, is a horrible lie.

To revert to the opening statement, when a young man goes out with the "fellows" to have a "devil of a time," that is usually the very thing that he succeeds in accomplishing, and it is not nearly always the young fellows either. When on such an excursion, the lower the dive the grosser the surroundings, the better they are satisfield. Under such conditions the demon of venereal poison finds easy victims.

On the other hand, should his sister side-step from the straight paths of continence and indulge in oblique sexuality, she is relatively decent about the matter; she seeks as her mate one in whom the elements of affection have a part; she seeks one whom she regards as a friend or a lover, and she does not encounter a fraction of the danger of bringing the lifelong horror of obscure venereal poison as her offering to the hymeneal altar as does the male. I am not justifying lapse from virtue; I am handling the conditions which exist, meeting what is and not what I would have. Consequently, I repeat, the bride has immeasurably a greater right to demand sexual purity of her spouse at the nuptial couch than he has to exact the like standard of virtue of her. We must not, because we can not, measure by Utopian desire. False ideals ever lead us farther and farther from truth.

The bestial animalism of the American negro has sounded the early doom of the race exactly through the elements, outlined in this paper, and the ravages of syphilis has placed the mercurial Frenchman in the vanishing class and of the progeny left to him, is spawning that hideous creature, the "Apache." Meanwhile, the scientific world is gingerly fooling with the question of the cause of the national French "race suicide," when statistics has answered that more than half of the adult Parisian males are syphilitic. The same insidious poison is at work in our own land. The two great exponents of Omnipotence have spoken.

Religion tells us that "The wages of sin is death"; evolution tells us of "the survival of the fittest." Whatever stands in the way of the great plan for the development of the race must be inexorably swept aside. It lies with the medical profession, which has the training and the knowledge to combat this monster.

I have found three types of men in the medical profession. There are the muck-rakers, not in the modern signification of that word, but with its classic definition from Bunyan, the fellow whose sole purpose is the raking together of dollars, no matter how dirty; the wooden-headed mechanic, mechanically working at the trade of medicine; and the true professional man, the high priest of purity, serving at the altar of clean, sound manhood and womanhood for the "healing of the nations." Whichever of these three extremes of type he may class with, it is to the doctor's vocational interest to call a halt to this scourge of perverted sexuality, and while doing it he should remember the dictum, "The laborer is worthy of his hire," and he should secure the price of his labor.—*Medical Herald.*

